# Incurable advanced salivary gland tumours: a retrospective analysis and peek into the perplexing clinical and molecular intricacies from a tertiary care centre in India

**DOI:** 10.3332/ecancer.2023.1602

**Published:** 2023-09-21

**Authors:** Bipinesh Sansar, Neha Singh, Anuj Gupta, Bal Krishna Mishra, Abhishek Sharma, Rahul Rai, Pooja Gupta, Akhil Kapoor

**Affiliations:** 1 Department of Medical Oncology, HBCH and MPMMCC, Varanasi 221005, India; 2 Department of Pathology, HBCH and MPMMCC, Varanasi 221005, India

**Keywords:** salivary gland tumour, adenoid cystic, mucoepidermoid, mammary analogue secretory, salivary duct

## Abstract

**Background:**

Salivary gland tumours are rare cancers with variable course and prognosis. There is a paucity of data, especially for the advanced stages.

**Materials and methods:**

This is a retrospective analysis carried out in our institute. All patients seeking treatment for incurable advanced salivary gland tumours from October 2018 to September 2022 were included. Relevant clinical data were collected and appropriate statistical analysis was applied.

**Results:**

30 patients were included in the analysis. The parotid gland was the most common site of origin (73%). Adenoid cystic carcinoma (ACC) and salivary duct carcinoma (SDC) were equally (37%) the most common pathological subtypes. The majority of patients were males (73%) and lungs (57%) were the most common site of metastases. On molecular analysis, SDC had high rates of androgen receptor (AR) (90%) and human epidermal growth factor receptor 2 (HER2) (55%) positivity. Mucoepidermoid carcinoma (MEC) had AR and HER2 positivity rates of 17% and 20%, respectively, while for ACC it was even lower. A variety of treatment regimens including hormonal therapy, anti-HER2 targeted therapy and chemotherapy were used in first-line treatment. With an overall response rate (ORR) of 10/21 (48%), only 9/21 (43%) went on to receive second-line treatment with an ORR of 4/9 (44%). The progression-free survival (PFS) with first-line treatment (PFS1) was a median of 5 months. The median PFS1 was worst for MEC. The median overall survival (OS) was 10 months. Median OS for ACC, SDC and MEC were 11, 10 and 7 months, respectively. At 24 months, ACC had much higher survival (50%) than others (10%) indicating a proportion of ACC with an indolent course.

**Conclusion:**

Our analysis highlights the variable disease biology of advanced salivary gland tumours and throws light on the various possible treatment targets and strategies. Molecular profiling and advancement in targeted therapies are expected to increase survival in this group of rare cancers.

## Background

Salivary gland tumours are relatively rare cancers with incidence rates of 11.95 per 1,000,000 person-years [1, 2]. Although collectively termed together, their histology varies along with the presence of a gamut of molecular alterations which also differ based on the subtype [3, 4]. Their significant proportion are cured by surgery alone, while a few require definitive or adjuvant radiation therapy. Adjuvant systemic therapy is not recommended currently. This strategy leads to reasonably acceptable 5-year overall survival (OS) rates of 55%–85% [1]. However, sometimes these tumours may present upfront with locally advanced or metastatic disease or may recur with local and/or distant metastases which are not amenable to definitive treatment [5, 6].

These advanced salivary gland tumours consisting of stages 4A to 4C have a wide variation in their clinical behaviour ranging from indolent to highly aggressive disease courses. Also, the multitude of molecular alterations may affect the tempo of the disease and the responses to treatment [7].

Due to the rarity of these tumours, there is scarce data regarding real-life evidence of how the treatment pans out for these patients. Also, the limited number of randomised clinical trials for evaluating the efficacy of various therapeutic options leads to significant variations in clinical practice [8]. This has led to a lack of information on the number of patients who undergo molecular testing, those who receive treatment and the type of treatment received. Furthermore, there is a lack of clarity on how the various available treatments are sequenced and how it affects the outcome of these patients. For example, it is unknown whether initiating hormonal therapy as the first-line treatment is better than chemotherapy in androgen receptor (AR) positive cancers. Most of the time, the approach to chemotherapy is extrapolated from the guidelines for other head and neck cancers which have a different biology [6]. Thus, it is imperative that we know which treatment regimens are most effective in terms of responses elicited and how the prognosis in terms of survival varies with the current treatment patterns. The aim and purpose of our analysis is to summarise data from our country in terms of molecular tests done, various chemotherapeutic, hormonal and targeted treatment received and their effect on the outcome and comparison of the same with published data. We hypothesise a change in the treatment pattern in our country compared to other available data due to a paucity of widespread molecular testing for all patients.

## Materials and methods

This is a retrospective analysis carried out in the Medical Oncology Department of our institute. All patients seeking treatment for advanced salivary gland tumours from the period of October 2018 to September 2022 were included. The data were extracted from the electronic medical records by searching for the following keywords – ‘salivary gland tumours’, ‘adenoid cystic carcinoma’, ‘mucoepidermoid carcinoma’, ‘salivary duct carcinoma’ and ‘mammary analog secretory carcinoma’. Only advanced cases deemed to be unsuitable for curative treatment were selected for the analysis. Patients who were diagnosed at our centre but were not initiated on treatment or who did not complete at least 2 months of treatment were excluded. All the relevant clinical details like the duration and symptoms of presentation were noted. Past treatment details including the clinical stage and modalities of treatment administered were also noted. The relevant details post progression to advanced disease were studied to know the sites of progression and the treatment path adopted. For radiological response assessment, the Response Evaluation Criteria in Solid Tumours (RECIST) 1.1 criteria was used. For calculating overall response rates (ORRs), both partial response and stable disease as per RECIST criteria were included [9]. All the pathological details were noted including the molecular tests done and their interpretation. The molecular tests done included immunohistochemical (IHC) assays for AR and human epidermal growth factor receptor 2 (HER2) and next-generation sequencing (NGS) of tumour tissue wherever the results were available. NGS was done on SOPHiA Solid Tumour Plus Solution, which uses both DNA and RNA extracts. The outcome variables for our analysis were OS and progression-free survival (PFS). OS was calculated from the date of diagnosis till death. PFS was calculated from the date of the start of the therapy to the date of frank clinical or radiological progression. Patients lost to follow-up were contacted telephonically to know about their disease status. Those patients who could still not be traced in this manner were censored for the events of progression or death at an interval of 3 months post-last follow-up.

### Statistical analysis

The statistical analysis was done using the Statistical Package for Social Sciences version 20. Mean, median and mode were calculated for quantitative data. The normality of data were assessed by using Q–Q plots. Univariate analysis was done to derive the *p*-value by chi-square calculation. OS and PFS curves were derived by Kaplan–Meier method. A *p*-value cut-off of less than 0.05 was considered significant.

## Results

A total of 33 cases were identified. Out of these, one patient did not take any treatment at our centre, and for two patients follow-up details could not be traced after treatment initiation. So, these were excluded from the analysis. The basic clinical–pathological details are presented in Table 1.

The age range of patients was 25–82 years with a median of 52 years. The parotid gland was the most common site of origin (73%) while adenoid cystic carcinoma (ACC) and salivary duct carcinoma (SDC) were equally (37%) the most common pathological subtypes. The univariate analysis of various factors with the pathological subtypes is presented in Table 2. A male predominance (73%) was noted overall. Advanced mucoepidermoid carcinoma (MEC) was seen only in males while for advanced SDC, all except one patient was female. On the other hand, advanced ACC had a female predominance (55%). The majority of advanced tumours were recurrent (57%) rather than upfront advanced (43%). Among subtypes, ACC had the highest proportion (82%) of recurrent cases while other subtypes had a high proportion of upfront advanced cases. Lungs (57%) were the most common site of metastases, followed by bones. SDC (82%) and ACC (64%) had a high proportion of lung metastases but MEC had no incidence of the same.

The molecular profile of all tumours was analysed and the salient features are shown in Table 2. The AR and HER2 positivity rates are highest in SDCs while lowest in ACCs. The single case of Mammary analogue secretory carcinoma (MASC) was positive for pan-TRK IHC.

NGS was done in seven patients. One patient with SDC had mutations in the PIK3CA, HRAS and TP53 genes concurrently.

The treatment profile of all the patients is provided in the Supplementary Table 1. A variety of treatment regimens were used in first-line treatment of 21 patients who were started on systemic therapy. Four asymptomatic patients were kept on observation. The most common chemotherapy regimen was a combination of paclitaxel and carboplatin with or without trastuzumab based on the HER2 status, while the most common hormonal therapy was the combined androgen blockade (CAB) of bicalutamide and leuprolide. The ORR to first-line treatments was 10/21 ( 48%). Only 9/21 (43%) went on to receive second-line treatment in which the ORRs were 4/9 (44%). The best response to first-line therapy was seen in ACC (75%) while MEC had zero response rates.

**Figure 1.**
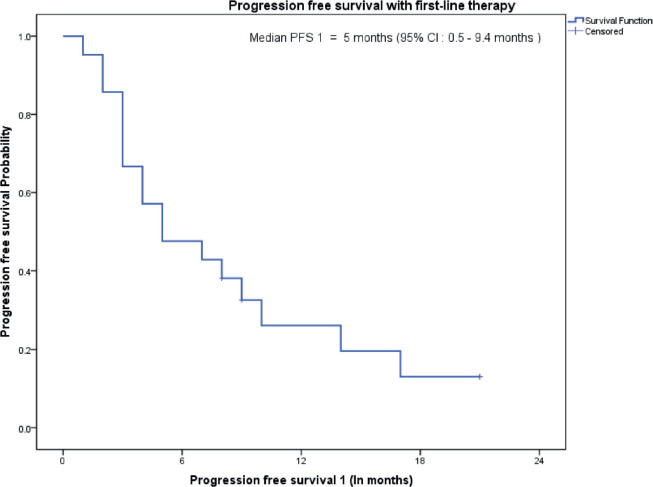
PFS on first-line treatment.

**Figure 2.**
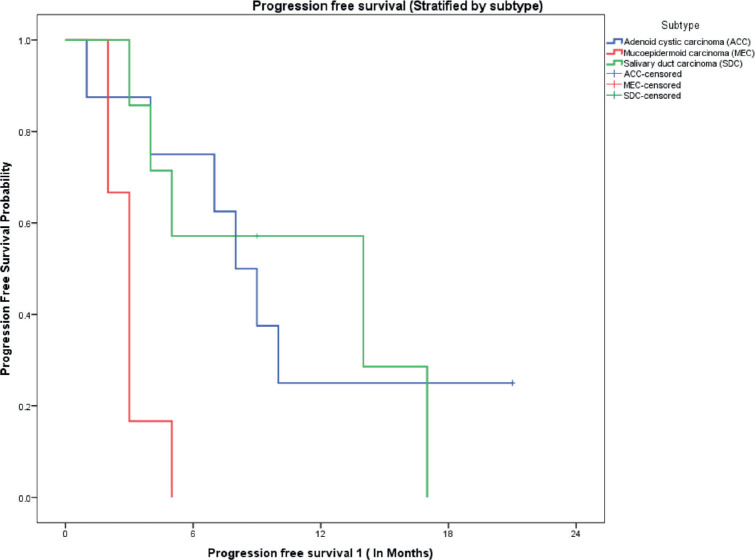
PFS on first-line therapy stratified by pathological subtypes.

**Figure 3.**
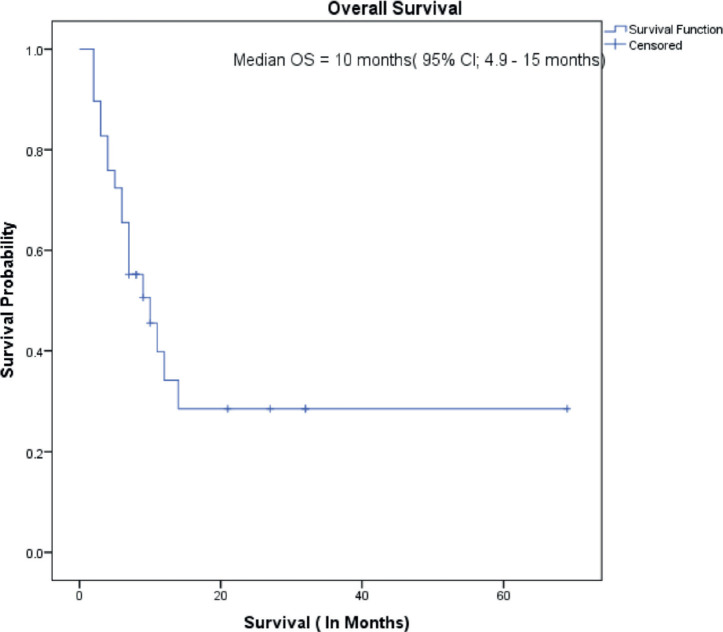
Overall survival.

**Figure 4.**
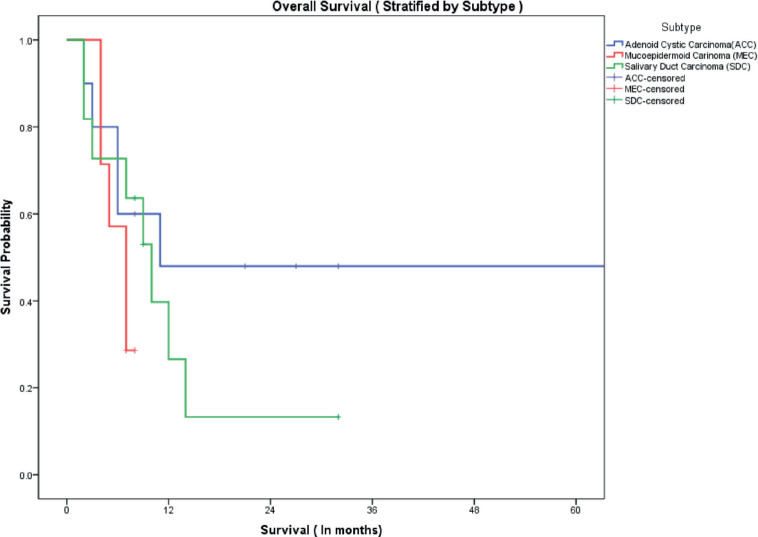
OS stratified by subtype.

The median follow-up of all patients was 18 months with the range being 6–69 months. The PFS with first-line treatment (PFS1) was a median of 5 months ( 95% confidence interval (CI); 0.5–9.4 months) as depicted in Figure 1. The median PFS1 was worst for MEC followed by ACC and SDC respectively as shown in Figure 2. After 17 months of follow-up, ACC had a better PFS1 than SDC indicating a subgroup with a better prognosis. The PFS with second-line treatment (PFS2) was a median of 4 months (95% CI; 2.5–5.4 months). The median OS was 10 months ( 95% CI; 4.9–15 months) depicted in Figure 3. Median OS for ACC, SDC and MEC were 11, 10 and 7 months, respectively, as depicted in Figure 4. ACC has the best survival while MEC has the worst. At 24 months, ACC had much higher survival (50%) than others (10%).

## Discussion

We would like to restate our aim of bringing out the clinical, pathological, molecular and treatment heterogeneity of advanced salivary gland tumours and the patterns observed at a tertiary care centre in our country. There is a paucity of data focussing on this group of patients in advanced stages [10]. Our study adds value by providing important inputs relevant to the treatment in the current era of molecular profiling.

The ACC group had the maximum percentage of recurrent cases (82%). This might be due to their indolent nature and long natural history leading to more patients being detected at early resectable stages. This will lead to more chances of them undergoing definitive surgeries and later presenting with recurrence. On the other hand, the SDC and MEC groups present more commonly with upfront unresectable or metastatic disease due to their aggressive tempo [7].

There is a lack of consensus on the first-line treatment option to be used as evidenced by the use of as many as seven different regimens. Also, some patients may be mildly symptomatic or asymptomatic at presentation for whom observation may be a plausible option [7]. The PFS of just 5 months on first-line treatments along with a less than 50% ORR indicates that the disease is not very sensitive to chemotherapy and targeted treatments and may progress rapidly indicating the complexity of underlying driver pathways [11]. However, 43% of patients were able to receive second-line therapies, thus suggesting that disease biology in this subset of patients is favourable leading to an indolent course. Again, a wide variety of second-line regimens were used. Some of these treatment regimens were extrapolated from the head and neck treatment data.

Comparing our data on ACC with other data available from meta-analyses and other studies, some similarities are appreciated [12]. The ORR with platinum-based combinations was in the range of 50%–80% compared to our data of 75% [13–16]. Tyrosine kinase inhibitors like lenvatinib, sunitinib, axitinib and sorafenib were associated with high rates of stable disease 60%–90% [17–21]. Our patients treated on lenvatinib and sorafenib also demonstrated high disease control rates even in the third-line setting. This suggests that these agents can be used from the second line onward and even may be a suitable maintenance strategy post-first-line taxane-platinum combination therapy. Also, watchful observation seems to be an excellent option in asymptomatic patients [22]. However. the median OS was only 11 months despite a 2-year survival of 50% indicating the need to identify this subset of aggressive ACCs [23]. Although the numbers are small, female patients in our dataset had the most aggressive disease (progression within 6 months of treatment initiation) and may warrant more potent therapy [24].

SDCs having near universal AR and very high HER2 positivity rates respectively are seen even in our patients [25–28]. The response rates in a systematic review of these patients were 60%–70%,18%–53% and 10%–50% with HER2 targeted therapy, androgen blockade therapy, and chemotherapy respectively [29–34]. Our response rates are similar with a 57% response rate. Although CAB was not very effective in our patients, a reasonable conclusion cannot be reached due to the low sample size. The available data regarding PFS 1 and OS is scarce but our patients had a median PFS1 and OS comparable with those with ACC. But, the 2-year survival of less than 20% indicates aggressiveness. As discussed by Dalin* et al* [26], the molecular signature similarities with apocrine breast cancer may be extrapolated in SDC with reference to future research in treatment options [26]. Thus, HER2-directed therapy combined with chemotherapy followed by CAB and anti-HER2 therapy as maintenance seems to be a sound strategy.

MECs had the poorest response to treatment and equally poor survival. This, along with the lack of adequate data, calls for more research on molecular niches and effective treatment strategies [35–37]. We found a case of MASC which was positive for IHC for pan-TRK. This IHC was utilised in line with the available literature suggesting the benefits of pan-TRK IHC as a time and tissue-efficient screen for NTRK fusions [38]. Recently, the European Society of Medical Oncology has released guidelines for the management of salivary gland tumours emphasising molecular testing [39]. A report by Kapoor *et al* [10] also highlighted the importance of identifying molecular targets and proposed a treatment algorithm for this rare disease. So, it is expected that a NGS-based testing strategy will soon become the standard of care.

## Conclusion

Our analysis highlights the variable disease biology of advanced salivary gland tumours, especially the indolent nature of ACC compared to others. It also throws light on the various possible treatment targets and strategies like CAB and its combination with anti-HER2 therapy. Molecular profiling and advancement in targeted therapies are expected to increase the survival in this group of rare cancers by enabling a more personalised treatment approach.

## Conflicts of interest

The authors report no conflicts of interest.

## Data sharing

The data that support the findings of this study are available from the corresponding author, upon reasonable request.

## Funding

None of the co-authors have received any financial aid in carrying out this analysis and neither do they have any conflicting financial or ethical interests with reference to this manuscript.

## Figures and Tables

**Table 1.** Clinical–pathological details of patients included in the analysis.

Sex	Males	22/30 (73%)
	Females	8/30 (27%)
Tumour primary site	Parotid gland	22/30 (73%)
	Submandibular gland	5/30 (17%)
	Sublingual and minor salivary glands	3/30 (10%)
Tumour histopathology	ACC	11/30 (37%)
	SDC	11/30 (37%)
	MEC	7/30 (23%)
	MASC	1/30 (3%)
Disease stage at presentation	Recurrent advanced	17/30 (57%)
	Upfront advanced	13/30 ( 43%)
Metastatic sites	Lungs	17/30 (57%)
	Bone	7/30 (23%)

**Table 2.** Univariate analysis for association of various clinical factors with different subtypes of salivary gland tumours.

Tumour type	ACC	SDC	MEC	MASC	*p*-value (chi-square method)
Male sex	5/11 (45%)	10/11 (91%)	7/7 (100%)	0/1 (0%)	0.01
Female sex	6/11 (55%)	1/11 (9%)	0/7 (0%)	1/1 (100%)	0.02
Upfront advanced disease	2/11 (18%)	6/11 (55%)	5/7 (71%)	0/1 (0%)	0.09
Recurrent disease	9/11 (82%)	5/11 (45%)	2/7 (29%)	1/1 (100%)	0.06
Lung metastases	7/11 (64%)	9/11 (82%)	0/7 (0%)	1/1 (100%)	0.04
AR	1/9 (11%)	9/10 (90%)	1/6 (17%)	0/1 (0%)	0.001
HER2	0/8 (0%)	6/11 (55%)	1/5 (20 %)	0/1 (0%)	0.031
Pan TRK	NA	NA	NA	1/1(100%)	
Response rate to first-line therapy	6/8 (75%)	4/7 (57%)	0/6 (0%)	NA	0.017

**Supplementary Table 1.** Treatment details of all patients.

Number	Age/sex	Primary site and type	AR	HER2	First line	Second line	Third line	Outcome
1	46/M	ACC, Parotid	-	-	Obs	NA	NA	Alive
2	54/F	ACC, Parotid	Neg	Neg	P C Cycle1	NA	NA	Death
3	68/F	ACC, Parotid	Neg	Neg	SA Gem	OMCT	NA	Lost to follow-up
4	66/F	ACC, Parotid	Neg	Neg	BSC	NA	NA	Death
5	29/F	ACC, Parotid	-	-	CAB	NA	NA	Death
6	51/F	ACC, Parotid	Neg	-	Obs	P C f/b OMCT Maint	NA	Alive
7	54/M	ACC, SMG	Neg	Neg	Pall RT	NA	NA	Death
8	35/F	ACC, Sublingual	Neg	Neg	P C	P C Rechallenge	Lenvatinib	Alive
9	40/M	ACC, Minor salivary gland	Neg	Neg	P C	P C	NA	Alive
10	40/M	ACC, Parotid	Neg	Neg	CAP	P C	Lenvatinib	Alive
11	28/M	ACC, Minor salivary gland	Neg	Neg	Sorafenib	Enzalutam-ide	P C	Alive
12	60/M	SDC, SMG	Pos	Pos	Pall RT	NA	NA	Death
13	35/F	SDC, Parotid	-	Pos	Pall RT	NA	NA	Lost to follow-up
14	72/M	SDC, Parotid	Pos	Neg	Pall RT	NA	NA	Death
15	58/M	SDC, Parotid	Pos	Neg	P C	CAB	NA	Death
16	65/M	SDC, Parotid	Neg	Pos	P C Trastu	NA	NA	Death
17	42/M	SDC, Parotid	Pos	Pos	P C	NA	NA	Alive
18	82/M	SDC, Parotid	Pos	Neg	CAB	NA	NA	Alive
19	58/M	SDC, Parotid	Pos	Neg	BSC	NA	NA	Death
20	44/M	SDC, Parotid	Pos	Pos	Bicalutamide	NA	NA	Death
21	62/M	SDC, Parotid	Pos	Pos	P C Trastu	CAB	NA	Alive
22	52/M	SDC, Parotid	Pos	Neg	CAB	P C	NA	Lost to follow-up
23	61/M	MEC, Parotid	Pos	Pos	P C	P C Trastu + Leuprolide	NA	Death
24	46/M	MEC, Parotid	Neg	Neg	CAP	NA	NA	Death
25	35/M	MEC, SMG	-	-	Obs	NA	NA	Alive
26	67/M	MEC, Parotid	Neg	Neg	P C	NA	NA	Death
27	25/M	MEC, Parotid	Neg	Neg	Doce Cis	Pall RT	NA	Death
28	81/M	MEC, SMG	Neg	-	LD Nivo + OMCT	NA	NA	Death
29	58/M	MEC, SMG	Neg	Neg	CAP	Paclitaxel	NA	Lost to follow-up
30	38/F	MASC, Parotid	NTRK 1 Pos		Obs	NA	NA	Alive

ACC: adenoid cystic carcinoma, AR: androgen receptor, BSC: best supportive care, CAB: combined androgen blockade, CAP: cyclophosphamide, adriamycin, cisplatin, Doce cis: docetaxel plus cisplatin, F: female, HER2: human epidermal growth factor receptor 2, LD Nivo: low dose Nivolumab, M: male, MASC: mammary analogue secretory carcinoma, MEC: muco-epidermoid carcinoma, NA: not applicable, Neg: negative, NTRK: neurotrophic tyrosine receptor kinase, Obs: observation, OMCT: oral metronomic chemotherapy, P C: paclitaxel plus carboplatin, Pall: palliative, Pos: positive, RT: radiotherapy, Trastu: trastuzumab, SDC: salivary duct carcinoma, SMG: submandibular gland.

## References

[1] Carvalho AL, Nishimoto IN, Califano JA (2005). Trends in incidence and prognosis for head and neck cancer in the United States: a site-specific analysis of the SEER database. Int J Cancer.

[2] Boukheris H, Curtis RE, Land CE (2009). Incidence of carcinoma of the major salivary glands according to the WHO classification, 1992 to 2006: a population-based study in the United States. Cancer Epidemiol Biomarkers Prev.

[3] Iyer J, Hariharan A, Cao UMN (2021). An overview on the histogenesis and morphogenesis of salivary gland neoplasms and evolving diagnostic approaches. Cancers.

[4] Bobati SS, Patil BV, Dombale VD (2017). Histopathological study of salivary gland tumors. J Oral Maxillofac Pathol.

[5] Wang X, Luo Y, Li M (2017). Management of salivary gland carcinomas – a review. Oncotarget.

[6] Geiger JL, Ismaila N, Beadle B (2021). Management of salivary gland malignancy: ASCO guideline. J Clin Oncol.

[7] Son E, Panwar A, Mosher CH (2018). Cancers of the major salivary gland. J Oncol Pract.

[8] Mizrachi A, Bachar G, Unger Y (2017). Submandibular salivary gland tumors: clinical course and outcome of a 20-year multicenter study. Ear Nose Throat J.

[9] Ruchalski K, Braschi-Amirfarzan M, Douek M (2021). A primer on RECIST 1.1 for oncologic imaging in clinical drug trials. Radiol Imaging Cancer.

[10] Kapoor A, Noronha V, Chougule A (2020). Molecular tumor board: case 4 salivary gland cancer. Cancer Res Stat Treat.

[11] Yousaf A, Sulong S, Abdullah B (2022). Heterogeneity of genetic landscapes in salivary gland tumors and their critical roles in current management. Medeni Med J.

[12] Ko JJ, Siever JE, Hao D (2016). Adenoid cystic carcinoma of head and neck: clinical predictors of outcome from a Canadian centre. Curr Oncol.

[13] Airoldi M, Fornari G, Pedani F (2000). Paclitaxel and carboplatin for recurrent salivary gland malignancies. Anticancer Res.

[14] Licitra L, Cavina R, Grandi C (1996). Cisplatin, doxorubicin and cyclophosphamide in advanced salivary gland carcinoma. A phase II trial of 22 patients. Ann Oncol.

[15] Nakano K, Sato Y, Sasaki T (2016). Combination chemotherapy of carboplatin and paclitaxel for advanced/metastatic salivary gland carcinoma patients: differences in responses by different pathological diagnoses. Acta Otolaryngol.

[16] Schramm VL, Srodes C, Myers EN (1981). Cisplatin therapy for adenoid cystic carcinoma. Arch Otolaryngol.

[17] Ho AL, Dunn L, Sherman EJ (2016). A phase II study of axitinib (AG-013736) in patients with incurable adenoid cystic carcinoma. Ann Oncol.

[18] Hotte SJ, Winquist EW, Lamont E (2005). Imatinib mesylate in patients with adenoid cystic cancers of the salivary glands expressing c-kit: a Princess Margaret Hospital phase II consortium study. J Clin Oncol.

[19] Chau NG, Hotte SJ, Chen EX (2012). A phase II study of sunitinib in recurrent and/or metastatic adenoid cystic carcinoma (ACC) of the salivary glands: current progress and challenges in evaluating molecularly targeted agents in ACC. Ann Oncol.

[20] Tchekmedyian V, Sherman EJ, Dunn L (2019). Phase II study of lenvatinib in patients with progressive, recurrent or metastatic adenoid cystic carcinoma. J Clin Oncol.

[21] Thomson DJ, Silva P, Denton K (2015). Phase II trial of sorafenib in advanced salivary adenoid cystic carcinoma of the head and neck. Head Neck.

[22] Lorini L, Ardighieri L, Bozzola A (2021). Prognosis and management of recurrent and/or metastatic head and neck adenoid cystic carcinoma. Oral Oncol.

[23] Cantù G (2021). Adenoid cystic carcinoma. An indolent but aggressive tumour. Part B: treatment and prognosis. Acta Otorhinolaryngol Ital.

[24] Marcinow A, Ozer E, Teknos T (2014). Clinicopathologic predictors of recurrence and overall survival in adenoid cystic carcinoma of the head and neck: a single institutional experience at a tertiary care center. Head Neck.

[25] Boon E, Bel M, Boxtel W (2018). A clinicopathological study and prognostic factor analysis of 177 salivary duct carcinoma patients from The Netherlands. Int J Cancer.

[26] Dalin MG, Desrichard A, Katabi N (2016). Comprehensive molecular characterization of salivary duct carcinoma reveals actionable targets and similarity to apocrine breast cancer. Clin Cancer Res.

[27] Takase S, Kano S, Tada Y (2017). Biomarker immunoprofile in salivary duct carcinomas: clinicopathological and prognostic implications with evaluation of the revised classification. Oncotarget.

[28] Schmitt NC, Kang H, Sharma A (2017). Salivary duct carcinoma: an aggressive salivary gland malignancy with opportunities for targeted therapy. Oral Oncol.

[29] Corrêa TS, Matos GDR, Segura M (2018). Second-line treatment of HER2-positive salivary gland tumor: ado-trastuzumab emtansine (T-DM1) after progression on trastuzumab. Case Rep Oncol.

[30] Viscuse PV, Price KA, Garcia JJ (2019). First line androgen deprivation therapy vs. chemotherapy for patients with androgen receptor positive recurrent or metastatic salivary gland carcinoma – a retrospective study. Front Oncol.

[31] Uijen MJM, Lassche G (2020). Systemic therapy in the management of recurrent or metastatic salivary duct carcinoma: a systematic review. Cancer Treat Rev.

[32] Takahashi H, Tada Y, Saotome T (2019). Phase II trial of trastuzumab and docetaxel in patients with human epidermal growth factor receptor 2-positive salivary duct carcinoma. J Clin Oncol.

[33] Locati LD, Perrone F, Cortelazzi B (2016). Clinical activity of androgen deprivation therapy in patients with metastatic/relapsed androgen receptor-positive salivary gland cancers. Head Neck.

[34] Fushimi C, Tada Y, Takahashi H (2018). A prospective phase II study of combined androgen blockade in patients with androgen receptor-positive metastatic or locally advanced unresectable salivary gland carcinoma. Ann Oncol.

[35] Nakano T, Yamamoto H, Hashimoto K (2013). HER 2 and EGFR gene copy number alterations are predominant in high‐grade salivary mucoepidermoid carcinoma irrespective of MAML 2 fusion status. Histopathology.

[36] Ullah A, Khan J, Waheed A (2023). Mucoepidermoid carcinoma of the salivary gland: demographics and comparative analysis in U.S. children and adults with future perspective of management. Cancers.

[37] Wang K, McDermott JD, Schrock AB (2017). Comprehensive genomic profiling of salivary mucoepidermoid carcinomas reveals frequentBAP1, PIK3CA, and other actionable genomic alterations. Ann Oncol.

[38] Hechtman JF, Benayed R, Hyman DM (2017). Pan-Trk immunohistochemistry is an efficient and reliable screen for the detection of NTRK fusions. Am J Surg Pathol.

[39] van Herpen C, Vander Poorten V, Skalova A (2022). Salivary gland cancer: ESMO-European Reference Network on Rare Adult Solid Cancers (EURACAN) clinical practice guideline for diagnosis, treatment and follow-up. ESMO Open.

